# Role of neutrophils in acute viral infection

**DOI:** 10.1002/iid3.500

**Published:** 2021-09-02

**Authors:** Yuan Ma, Yue Zhang, Liuluan Zhu

**Affiliations:** ^1^ Institute of Infectious Diseases Peking University Ditan Teaching Hospital Beijing China; ^2^ Beijing Key Laboratory of Emerging Infectious Diseases, Institute of Infectious Diseases, Beijing Ditan Hospital Capital Medical University Beijing China

**Keywords:** antiviral immunity, COVID‐19, neutrophil, neutrophil extracellular trap

## Abstract

Neutrophils play multiple roles in acute viral infections. They restrict viral replication and diffusion through phagocytosis, degranulation, respiratory burst, secretion of cytokines, and the release of neutrophil extracellular traps, as well as, activate the adaptive immune response. However, the overactivation of neutrophils may cause tissue damage and lead to poor outcomes. Additionally, some characteristics and functions of neutrophils, such as cell number, lifespan, and antiviral capability, can be influenced while eliminating viruses. This review provides a general description of the protective and pathological roles of neutrophils in acute viral infection.

## 1 INTRODUCTION

Infectious viral diseases are a major problem for human health, especially some of the newly emerging viruses that seriously threaten human life and health, such as the highly pathogenic avian influenza virus, Ebola virus, Middle East respiratory syndrome (MERS)‐coronavirus, and the severe acute respiratory syndrome coronavirus 2 (SARS‐CoV‐2).^1^, ^2^, ^3^ The global outbreak of the coronavirus disease in 2019 (COVID‐19) caused by SARS‐CoV‐2 threatened global public health with high morbidity and mortality.^4^ In viral infections, neutrophils help in resisting infections and provide immunity, which may result in severe tissue injuries and poor outcomes. In this article, we have reviewed the current literature regarding the protective and pathological roles of neutrophils in viral infections, with particular attention to the studies related to respiratory diseases.

Neutrophils, also known as polymorphonuclear leukocytes, develop from blast cells, mature into terminally differentiated cells in the bone marrow, and are released into the peripheral blood. Under physiological conditions, the turnover rate of neutrophils is 10^11^ per day, which is affected by cell death and production rate.^5^ Neutrophils are relatively short‐lived. It was thought that neutrophils have a lifespan of 8–12 h in the peripheral blood. An in vivo study in humans showed that under homeostatic conditions, the average circulating life of neutrophils is 5.4 days.^6^ The rate of generation and the lifespan of neutrophils are further increased during inflammation or infection, as reviewed below. The role of neutrophils in fighting bacterial pathogens has been well‐documented; their role in viral infections has gained increasing attention in recent years and needs to be well‐documented.^7^

### 1.1 Neutrophils recognize viruses and virus‐infected cells and infiltrate into the sites of infection

At the onset of a viral infection, neutrophils are rapidly recruited at the site of infection from the blood and are mobilized to differentiate and migrate out of the bone marrow. In the respiratory infections caused by influenza, MERS, and respiratory syncytial viruses (RSV), neutrophils were found to infiltrate into the lungs in large numbers.^8^, ^9^, ^10^ In children with RSV‐induced bronchiolitis, neutrophils account for about 80% of the infiltrating inflammatory cells.^9^ Neutrophils express chemotactic receptor 1(CXCR1) and anaphylatoxin complement 5a (C5a) receptor on the surface of the cell membrane, which specifically binds to the chemokine (C‐X‐C motif) ligand 8 (CXCL8) and C5a, respectively, and mediate the infiltration of the neutrophils to the sites of infection.^11^, ^12^, ^13^ In RSV infection, neutrophils move to the infected sites and migrate across the human nasal respiratory epithelium through para and transcellular migration.^14^, ^15^ Influenza virus‐infected human alveolar epithelial cells secret CXCL8 and granulocytemacrophage‐colony stimulating factor (GM‐CSF) to attract and activate neutrophils.^16^ The degree of neutrophil infiltration is related to the viral load, viral strain type, and disease severity.^17^

Neutrophils can recognize pathogen‐associated molecular patterns (PAMPs) on viruses and initiate signaling cascades to induce innate immune response using multiple pattern recognition receptors (PRRs) on the cell surface.^18^, ^19^ Toll‐like receptors (TLRs) are classic PRRs that detect viral proteins and nucleic acids.^20^ TLR3, TLR7, TLR8, and TLR9 are located in the intracellular compartment and are dedicated to virus detection and nucleic acid recognition, in which TLR3 recognizes double‐stranded RNA, TLR7 and TLR8 recognize single‐stranded viral RNA, and TLR9 recognizes unmethylated CPG DNA of the virus.^21^ Notably, TLR3 and TLR7 are not expressed on human neutrophils but are expressed and functional on mouse neutrophils.^22^, ^23^, ^24^ In coxsackievirus B3‐infected mice, neutrophils recognize viral ssRNA through cytoplasmic TLR8 rather than TLR7 to activate the nuclear factor‐κB (NF‐κB) signaling pathway and trigger inflammation.^25^ Human neutrophils are also able to directly bind to influenza A virus (IAV) through cell surface glycoproteins or glycolipids such as CD43.^26^ TLR4 participates in the antiviral defense against RNA viruses in innate immune cells by recognizing the fusion proteins of RSV.^27^ On the contrary, a study found that neutrophils were indirectly activated by the CXCL8 released from the RSV‐infected cells, rather than being activated directly by the RSV.^28^

### 1.2 The protective role of neutrophils in acute viral infection

#### 1.2.1 Phagocytosis

Neutrophils can engulf virions and the apoptotic bodies containing the virus, thereby promoting virus clearance and preventing viral replication in and infection of the surrounding cells.^29^, ^30^ In influenza‐infected mice, neutrophils reach the infected site of the lung and swallow viruses and the virus‐infected cells.^31^ Once phagocytosis occurs, neutrophils utilize potent antimicrobial substances or hydrolases to kill or inactivate pathogens.^32^ However, some studies have hypothesized that phagocytosis of viruses by neutrophils is not an active process; instead, viruses actively infect and spread to neutrophils.^7^

#### 1.2.2 Degranulation

Neutrophils are rich in many kinds of granules, divided into primary (or azurophilic) granules, secondary (or specific) granules, and tertiary granules. There are pre‐formed effector molecules in the neutrophil granules, which rapidly release the contents of the granules after infection. Studies have shown that RSV and dengue viruses stimulate neutrophils to degranulate the granule enzymes myeloperoxidase (MPO) and lactoferrin at the sites of infection.^33^, ^34^ Additionally, compared to healthy individuals, patients infected by COVID‐19 have drastically higher levels of azurophilic granules in their nasopharyngeal specimen, comprising MPO, elastase, and cathepsin G.^35^ MPO released by neutrophils can catalyze the reaction of halide with hydrogen peroxide (H_2_O_2_) and generate hypohalous acids, which have potent antiviral properties.^36^ Lactoferrin interacts with microorganisms, viruses, and the cell surface, thereby resisting microbial and viral adhesion and preventing entry into the host cell.^37^ Studies have shown that lactoprotein can bind to receptors on the coronavirus (e.g., SARS‐COV‐2) to prevent their entry.^38^ Other studies have shown that lactoferrin can reduce human norovirus infection, although the mechanism may be indirect and involve the innate interferon response.^39^

#### 1.2.3 Production of cytokines, chemokines, and antimicrobial agents

To defend against viruses and launch other immune cells, activated neutrophils can produce multiple cytokines, chemokines, and antimicrobial substances. In viral infections, activated neutrophils release pro‐inflammatory cytokines, such as tumor necrosis factor‐α (TNF‐α), IL‐6, IL‐8, and IFN, to recruit and activate more neutrophils and limit viral replication and disease progression.^7^ Cytokines produced by neutrophils limit the replication of viruses and prevent the development of severe diseases.^40^ Most of the antimicrobial substances have antibacterial properties, and some of them, such as matrix metalloproteinase‐9 (MMP9), cathelicidins, and α‐defensins, also possess antiviral properties.^7^ The activated neutrophils can release MMP9 to eliminate the influenza virus; in the same study, the authors found that MMP9 promotes infiltration of neutrophils into the lungs by degrading the extracellular matrix, which results in increased inflammation in the lungs.^17^ An in vitro study demonstrated that human α‐defensin‐1 released by neutrophils was able to inhibit influenza virus replication by blocking the life cycle of the virus.^41^ The defensins and cathelicidins in neutrophil granules are cationic antimicrobial peptides, which can neutralize IAV.^40^ Cathelicidins have anti‐influenza activity in vivo by reducing viral replication and inhibiting the production of inflammatory mediators.^40^ IAV infection, including the infection by the highly virulent H3N2 influenza virus, rapidly induces the type I interferon signaling pathway in neutrophils, thus limiting viral replication.^40^, ^42^, ^43^

#### 1.2.4 Respiratory burst

Neutrophils use nicotinamide adenine dinucleotide phosphate (NADPH) oxidase to catalyze the production of reactive oxygen species (ROS) as the primary molecule against viruses by a process called “respiratory burst” or “oxidative burst”.^44^ Several viruses, including IAV, herpes simplex virus (HSV), and RSV, have been shown to trigger neutrophils to produce ROS through mechanisms such as the NADPH oxidase system.^45^, ^46^, ^47^ ROS are highly oxidizing and effectively destroy the chemical groups and components necessary for viral nucleic acid replication and virus transmission, thus controlling the spread of viruses.^7^, ^48^

#### 1.2.5 Neutrophil extracellular traps (NETs)

NETs are web‐like structures of nuclear chromatin coated with antimicrobial particles and cytoplasmic proteins, including histones, elastase, MPO, and α defensins.^49^, ^50^ There is some debate about the mechanism of formation of NETs. A popular hypothesis states that NETs are formed due to NETosis, a special form of programmed death of neutrophils. However, research by Yousefi et al. showed that short‐term TLR4 or C5a stimulation after GM‐CSF priming could make viable neutrophils generate NETs; the neutrophils contained mitochondrial DNA instead of nuclear DNA, and the process was ROS dependent.^51^ The same group later concluded that NETs are DNA scaffolds composed of mitochondrial DNA bound to granular proteins, which do not depend on cell death. On the contrary, NETosis is a process of neutrophil rupture where chromosomal DNA is released.^52^ NETs were initially considered to eliminate bacteria and now are confirmed to protect against many viral pathogens, including RSV, influenza virus, dengue virus, and even human immunodeficiency virus (HIV).^53^ In viral infections, NETs capture the viral particles through the NET structure and eliminate the virus through high local concentrations of MPO and defensins, or at least prevent the virus from spreading from the local area to other tissues.^54^ NETs can capture RSV, preventing them from entering into the target cells and, in turn, preventing infection.^55^ Both viruses and virus‐derived factors have strong inducing effects on NET production.^53^, ^56^ Microscopic observations have shown that NETs capture HIV and prevent the virus from spreading to other tissues; NET‐related MPO and α‐defensin inhibit the infectivity of HIV.^57^ Some studies have found that NETs have no irreplaceable roles in antiviral immunity, and the role of NET production in innate immunity might be overemphasized.^49^, ^58^

### 1.3 Pathological effects of neutrophils in viral infections

As mentioned above, neutrophils exert antiviral effects through various mechanisms to limit viral replication and expansion. However, the antiviral effect of neutrophils might cause tissue injury because of the low specificity and high quantity of neutrophil antimicrobial substances, causing a reverse effect in the host antiviral response. A systemic study revealed that early participation of neutrophils might lead to a destructive feedforward congenital inflammatory circuit responsible for the development of serious diseases following IAV infection in mice.^59^

#### 1.3.1 Toxic enzymes

Neutrophil granules contain a variety of enzymes; excessive release of granules can cause vascular leakage, pulmonary edema, and hypoxemia.^10^ As mentioned above, MMP9 released by neutrophils exacerbates pathological effects in a mouse model of influenza virus infection.^17^ In children with RSV‐induced bronchiolitis, the degree of neutrophilic inflammation is related to the severity of the disease. Regarding the pathological mechanism, it was shown that neutrophil elastase destroyed elastin fibers and also the organization of the lung tissue. Additionally, cathepsin G, elastase, and protease 3 secreted by neutrophils also contributed to vascular leakage, inflammation, and pathological changes.^9^, ^60^

#### 1.3.2 Oxidative damage

During RSV infection, earlier studies had shown that clearance of neutrophils reduced lung inflammation.^61^ Subsequent studies further revealed the pathological mechanisms. Neutrophils in the lungs undergo oxidative burst and produce a large amount of ROS, which oxidize biomolecules, damage host cellular structures, and promote lung injury.^9^, ^28^ Similarly, in influenza infection, exuberant neutrophils produce a large amount of ROS that damages the epithelial‐endothelial barrier and affects the elimination of IAV, and finally, leads to detrimental inflammation and increased morbidity in mice.^62^, ^63^, ^64^, ^65^

#### 1.3.3 NET‐related tissue injury

Although NETs play a beneficial role in antiviral host protection, excessive NETs can lead to collateral damage.^53^ The NETs released by neutrophils have a direct harmful effect on surrounding cells, such as the endothelial cells and hepatocytes in the liver.^53^ The harmful effects of NETs are related to their components. Proteases, the source of autoantigens, are known to trigger and promote inflammation.^66^ Histones and MPO are the main substances in NETs that mediate cytotoxicity and the destruction of lung tissues.^67^ Many hydrolases such as elastase, cathepsin, and serine protease contained in neutrophils also cause host damage. Additionally, the deposition of NETs induces autoimmunity and causes local tissue damage and organ dysfunction.^7^, ^68^ Since the components of NETs are antigens of autoimmune diseases, they can induce the production of autoantibodies. These autoantibodies form an immune complex with NETs and deposit in the kidneys, causing tissue damage.^56^ NETs in the alveoli, histones, and MMP9 cause pulmonary capillary damage and obstruct small blood vessels, which leads to lung damage.^10^ In IAV‐infected mice, increased neutrophil infiltration and the elevated NET levels are associated with the damage of alveolar structure.^69^ In severe cases of H7N9 and H1N1 infection, increased production of NETs causes damage to alveolar epithelial and vascular endothelial cells, which are some of the major pathological factors of severe pneumonia.^10^ In patients with severe H1N1, high levels of NETs in the circulation are related to poor prognosis, and elevated levels of NETs in bronchoalveolar lavage fluid are related to lung pathology.^70^ Hantavirus infection increases the formation of NETs in patients and anti‐nuclear antigen autoantibodies, which can explain the mechanism of hemorrhagic fever.^56^

#### 1.3.4 Stimulation of mucus production

Neutrophils can cause airway obstruction by inducing the formation of mucus.^9^ Neutrophil depletion in RSV‐infected mice resulted in decreased mucin expression and TNF‐α production.^71^ In BALB/c mice, intratracheal administration of TNF‐α induced the production of mucus‐related proteins and mucus in the trachea.^71^ Additionally, it was reported that the formation of NETs in the airways of RSV‐infected calves caused airway obstruction.^72^ The NETs in the airways also trapped mucus and caused further embolism.^72^ The extracellular DNA and oxidative stress caused by the release of ROS and MPO during NET formation in the human airways increase the viscoelasticity of mucus.^73^ In calves with severe RSV infection, extensive airway obstructions are related to mucus rich in NETs.^55^, ^74^ NETs can be used as targets for the therapy of respiratory obstruction in severe RSV diseases.^72^

### 1.4 Influence of viral infection on neutrophils

#### 1.4.1 Impact on the number of neutrophils

The number of neutrophils is regulated by complex interactions among proliferation, apoptosis, and differentiation processes.^75^ Since neutrophils are the first line of defense against a viral invasion, the number of neutrophils in the local microenvironment increases sharply following a viral infection. Notably, the number of neutrophils in the respiratory tract is positively correlated with the virulence and dose of the influenza virus.^76^ In certain viral infections, such as severe fever with thrombocytopenia syndrome virus (SFTSV), the number of neutrophils in circulation is decreased for some reason.^77^ For example, when neutrophils migrate to the infected tissues and undergo NETosis or apoptosis, the development, differentiation, maturation, and bone marrow mobilization of neutrophils might be affected by viruses, which might negatively affect the neutrophil homeostasis.

#### 1.4.2 Impact on the neutrophil lifespan

Under the stimulation of the inflammatory signals, the lifespan of the migrated neutrophils can be extended to support neutrophil effector functions.^78^ A study found that RSV can extend the lifespan of human neutrophils by delaying or inhibiting apoptosis.^75^, ^79^ During IAV infection in mice, the immune mediators IL‐6 and granulocyte‐colony stimulating factor (G‐CSF) can prolong the life span of lung neutrophils.^80^ On the contrary, other studies have suggested that viral infection can promote the apoptosis of neutrophils. For example, IAV accelerates the apoptosis of human neutrophils.^75^ The HIV and Simian immunodeficiency virus (SIV) also induce neutrophil apoptosis, which may increase the sensitivity of the body to bacterial infection due to the viral infection, and the degree of neutrophil apoptosis is positively correlated with the severity of the disease.^81^, ^82^

#### 1.4.3 Impact on neutrophil function

To survive longer in infected hosts, viruses have evolved several antiviral escape mechanisms. Viruses can change the ability of neutrophils to release ROS and effector proteins, affect migration and adhesion functions of neutrophils, and even induce neutrophil apoptosis. The dengue virus reduces NET production in human patients rather than stimulating it; the PMA‐induced release of NETs was shown to reduce by 80% in the presence of the dengue virus.^83^ Studies have confirmed that neutrophils cannot effectively remove bacteria after a viral infection which is mainly because neutrophil chemotaxis, phagocytosis, killing, and release of antimicrobial peptides are inhibited after viral infections; thus, resulting in reduced resistance and increased host susceptibility to fungi and bacteria.^84^ Studies have shown that infection by the influenza virus reduces the bactericidal ability of neutrophils in the lungs due to the reduced MPO activity and impaired digestion, and/or lethality of the secondary bacterial infection.^85^

### 1.5 Neutrophils interact with other immune cells

Recently, studies have focused on the interactions between neutrophils and other innate and adaptive immune cells. In viral infections, neutrophils act as antigen‐presenting cells to guide and regulate the antiviral response in the host.^86^, ^87^

#### 1.5.1 Neutrophils interact with monocytes and macrophages

Neutrophils and macrophages are derived from common hematopoietic progenitor cells and share common features in phagocytosis and antigen presentation.^88^ Mononuclear macrophages are important effector cells for the antiviral immune response and produce cytokines and chemokines.^89^ In a viral infection, neutrophils regulate the antiviral mechanisms of monocytes and macrophages through the following process. First, neutrophils express classical monocyte and macrophage chemoattractants, such as CCL2, CCL3, CCL19, and CCL20, or use granular proteins, such as antimicrobial peptides, to recruit monocytes and macrophages at the site of the infection.^90^, ^91^ Neutrophil granule proteins enhance the adhesion and exudation of inflammatory monocytes.^92^ Second, neutrophils express cytokines and chemokines, such as monocyte chemotactic protein, TNF‐α, and IL‐1α, to enhance the microbicidal activity of monocytes and macrophages.^90^, ^92^ Third, neutrophil‐derived antimicrobial peptides enhance the ability of monocytes to phagocytose and secrete antimicrobial substances.^92^ Fourth, NETs promote the maturation of monocytes and enhance the expressions of IL‐10 and IFN‐γ.^93^ The release of IL‐1β, mediated by NOD‐, LRR‐ and pyrin domain‐containing protein 3 (NLRP3) inflammasome, is a significant trait of macrophages. In influenza‐infected mice, neutrophils provide the second signal of NLRP3 activation and promote the release of IL‐1β from alveolar macrophages.^94^ This pathway is a key host defense mechanism and must be strictly controlled to limit immune pathology.^94^ Additionally, macrophages can also influence neutrophils. During the inflammatory clearance stage, macrophages engulf apoptotic neutrophils, causing a decrease in IL‐23 production by macrophages and IL‐17 secretion by T cells, thereby reducing the production of G‐CSF and neutrophils.^95^ Phagocytosis of apoptotic neutrophils induces macrophages to transform into M2‐like subtypes, thereby promoting tissue repair during the resolution of inflammation.^96^ Therefore, the cross‐talk between neutrophils and monocytes/macrophages influences the antiviral response and controls disease pathology.

#### 1.5.2 Neutrophils interact with adaptive immune cells

Neutrophils affect the adaptive immune cells in viral infections.^6^ Studies have shown that neutrophils secret two important B cell ligands, viz., B cell‐activating factor (BAFF) and a proliferation‐inducing ligand (APRIL), to modulate the survival, differentiation, and maturation of B cells.^97^, ^98^ This effect can also be achieved through antigen presentation, pathogen metastasis and elimination from lymph nodes, and regulation of T‐cell helper responses. Neutrophils enhance T‐cell responses by producing chemokines or presenting antigens, thereby directing CD4 or CD8 lymphocytes through homologous antigen recognition.^92^, ^99^ Neutrophils migrate to lymph nodes after capturing antigens peripherally, affecting antigen‐specific T cells and dendritic cells. NETs can reduce the activation threshold of T cells, promote their activation and enhance immune response.^62^ Neutrophils are recruited early in the airway during the influenza infection and leave traces of CXCL12‐rich chemokines in the migration pathway, which helps the migration of virus‐specific CD8^+^ T cells into the tissues.^100^ In mouse IAV infection, neutrophils can present viruses to CD8^+^ T cells to facilitate the recruitment, proliferation, and secretion of IFN‐γ in the effector CD8^+^ T cells to remove the virus.^55^, ^86^, ^100^ Neutrophils can also transport viruses to the bone marrow and lymph nodes, assisting the proliferation of CD8^+^ T cells and the production of memory T cells.^101^, ^102^ In mouse IAV infection, depletion of neutrophils can impair the degree of response and the production of cytokines and cytotoxic effector function of CD8^+^ T cells.^103^, ^104^ Neutrophils infected by IAV can activate CD8^+^ T cells as APC and enhance their antiviral function.^7^ Studies have shown that immune‐mediated tissue damage in severe influenza infection is related to T cells, and some neutrophils can inhibit T cells and reduce their pathological effects.^105^ Cytokines produced by T_H_17 cells (such as IL‐17, CXCL8, IFN‐γ, TNF, and GM‐CSF) facilitate the migration and activation of neutrophils and also extend their lifespan.^66^

### 1.6 Neutrophils and COVID‐19

COVID‐19 is a novel respiratory disease caused by the SARS‐COV‐2, and its typical clinical manifestation is a mild and moderate respiratory infection characterized by flu‐like symptoms.^106^ However, some patients develop pneumonia and respiratory failure, for example, acute respiratory distress syndrome (ARDS), sepsis, and multiorgan failure.^107^, ^108^, ^109^, ^110^ The hyperactive immune response of severe COVID‐19 patients mainly consists of overwhelming infiltration, abnormal activation, and significantly decreased granularity of neutrophils.^111^ The cytokine storm during severe COVID‐19 infection, represented by increased plasma concentrations of IL‐1β, G‐CSF, and TNF‐α, strongly promotes neutrophil activation and chemotaxis.^112^, ^113^

The number of circulating neutrophils in COVID‐19 patients increases, and it was positively correlated with the severity of the disease.^114^ Although NETs are beneficial in the host defense against pathogens, they may contribute to organ damage and mortality in COVID‐19.^109^ Clinical evidence has shown that the increased NET formation is correlated with the development of ARDS in COVID‐19 patients and is a potential biomarker of the disease progression.^115^ Neutrophil counts and NET levels dramatically increase in extreme cases and poor prognostic patients.^116^, ^117^ The inducing factor of NETosis, the soluble platelet‐derived factors, and the NET‐induced cytokines, IL‐6, and CXCL8, are significantly elevated in COVID‐19 patients.^115^ NETs that interact with platelets initiate a thrombo‐inflammatory cascade, which makes blood hypercoagulable, and ultimately leads to platelet deposition and thrombosis in COVID‐19 patients.^118^ Our team reported that excessive activation of the anaphylatoxin‐NET axis is related to thrombosis and disease progression in COVID‐19 patients.^119^ In contrast, a study by Hidalgo showed that there was no correlation between NETs and markers of thrombosis, such as D‐‐dimers.^120^ These findings indicate that the relationship between NET and thrombosis is very complicated. Thus, more research should be conducted to support the involvement of NETs in the pathology of COVID‐19, and drugs that target NETs, such as inhibitors of neutrophil elastase and PAD4, should be used more carefully.^121^ Physicians should pay more attention to the role of NETs in COVID‐19 to make the right decision at critical times.

The increase in the level of calprotectin is a sensitive and dynamic marker of neutrophil activation and is also observed in the circulation of COVID‐19 patients.^108^ High levels of calprotectin have been found in many types of infection and inflammatory diseases.^108^ Calprotectin participates in the activation of innate immune sensors and the thrombus‐inflammatory storm that occurs in COVID‐19 patients.^108^ Calprotectin interacts with neutrophils and platelets and promotes the formation of thrombosis in COVID‐19 patients.^108^ Many studies have emphasized the emergence of abnormal neutrophil subsets induced by COVID‐19 infection. Silvin et al.^122^ and Schulte‐Schrepping et al.^107^ showed that patients with severe COVID‐19 release large amounts of calprotectin, and immature neutrophils with immunosuppressive characteristics accumulate in their blood and lungs, suggesting emergency myelopoiesis.^107^, ^122^ Plasma levels of calprotectin and immature neutrophils are powerful biomarkers of the severity of the COVID‐19 infection, and therapies targeting calprotectin and neutrophil can be considered as a part of the individualized treatment for some COVID‐19 patients.^122^ In patients at early stages of COVID‐19 infection, the peripheral blood mononuclear cell fraction was found to contain surprisingly high levels of neutrophils, and the neutrophil populations showed changes in cell size and internal complexity, such as low‐density neutrophils (LDNs) and immune forms; these features might help in predicting the severity of the disease.^123^ A study by Morrissey et al.^124^ found that LDNs in COVID‐19 patients expressed moderate levels of CD16, and showed pro‐inflammatory gene signatures, platelet activation, spontaneous formation of NETs, enhanced phagocytic ability, and generation of cytokines.

## 2 CONCLUSION

Till now, detailed studies on neutrophils have been performed for influenza, COVID‐19, and RSV‐related bronchiolitis. Neutrophils not only defend against viruses by phagocytosis and NETosis but also play an unfavorable role in tissue and organ damage in certain viral diseases. However, the precise mechanism of action of neutrophils in antiviral immunity is poorly understood. The lack of information leads to an inability to accurately conduct clinical interventions to adjust the body to an appropriate antiviral immune response. Neutrophil, the most abundant leukocyte, has been shown to function in infectious and noninfectious diseases. A thorough understanding of the working mechanism of neutrophils will not only benefit the treatment of viral diseases but will also promote the study of neutrophils in other diseases.

## CONFLICT OF INTERESTS

The authors declare that there are no conflict of interests.

## AUTHOR CONTRIBUTIONS

Yuan Ma performed the literature search and wrote the manuscript. Yue Zhang edited the manuscript. Liuluan Zhu edited and revised the manuscript.
